# Orally administered fluorescein angiography for ultra-wide-field imaging: is a safe and effective modality across different age groups and fundus diseases?

**DOI:** 10.3389/fcell.2025.1733128

**Published:** 2026-01-08

**Authors:** Yuke Ji, Xinya Hu, Yuting Hu, Sisi Lai, Xiaofeng Lu, Xuan Li, Zixiao Liu, Shaochong Zhang, Weihua Yang, Xue Yao

**Affiliations:** 1 Shenzhen Eye Hospital, Jinan University, Shenzhen, Guangdong, China; 2 Shenzhen Eye Hospital, Shenzhen Eye Medical Center, Southern Medical University Shenzhen, Shenzhen, Guangdong, China; 3 Department of Ophthalmology and Optometry, Fujian Medical University, Fuzhou, Fujian, China

**Keywords:** fluorescein angiography, fundus diseases, intravenous FA, oral FA, ultra-wide-field imaging

## Abstract

**Purpose:**

To comprehensively evaluate the clinical utility, imaging performance, and safety of orally administered fluorescein angiography (oral FA) combined with an ultra-wide-field imaging system in the diagnosis and management of fundus disorders.

**Methods:**

This prospective study enrolled 382 patients (676 eyes) aged 4–83 years, comprising 164 females and 218 males. All participants underwent oral FA after ingesting a weight-based dose of fluorescein sodium. Anonymized peak-phase images were independently graded by four retina specialists using a standardized three-parameter scoring system. Images were classified as high, moderate, or poor quality based on total scores. Circulation times (first appearance time, detailed visualization time, and optimal visualization time) and adverse events were systematically recorded. Statistical analyses assessed differences in image quality and timing across age groups and disease categories.

**Results:**

Oral FA was successfully performed in all cases. Among 676 eyes, 662 (97.9%) were graded as high quality, 12 (1.8%) as moderate quality, and 2 (0.3%) as poor quality. Clinically useful images were obtained in 99.7% of cases. No significant differences in image quality or circulation times were observed across age groups. However, image quality was significantly higher in retinal degenerative diseases compared to retinal vascular diseases (*P* = 0.001), though both groups maintained diagnostically adequate scores. In addition, no significant differences in circulation times (first appearance time, detailed visualization time, and optimal visualization time) were observed among any disease groups. Mild adverse events (nausea, rash) occurred in only 2.1% of patients, with no severe reactions—even in six patients with prior intravenous FA (IVFA) allergy history.

**Conclusion:**

Oral FA is a well-tolerated and clinically effective imaging modality that produces high-quality, diagnostically reliable angiograms across all age groups and multiple retinal disease categories. Its non-invasive nature, excellent safety profile, and ability to visualize peripheral pathology support its use as a practical and valuable alternative to conventional IVFA, particularly in pediatric, needle-phobic, or allergy-prone populations.

## 1 Introduction

Fundus diseases represent a leading cause of blindness worldwide (Li et al., 2023; He et al., 2023) The comprehensive evaluation of these conditions—encompassing retinal vascular diseases, chorioretinitis, retinal degenerative diseases, macular diseases, and optic nerve disorders—is crucial for accurate diagnosis, management, and monitoring of disease progression (Noh et al., 2023; Yu et al., 2025; Ji et al., 2023). Fluorescein angiography (FA) has been widely regarded as the gold standard imaging modality for assessing retinal and choroidal circulation (Ahmed et al., 2021; Hans et al., 2022). Conventional FA, which involves intravenous FA (IVFA), provides dynamic visualization of blood flow, vascular leakage, and pathological changes (Bulson and Faridi, 2017; Chen et al., 2022). However, IVFA is associated with potential side effects ranging from transient nausea and vomiting to rare but severe anaphylactic reactions (Ebrahimiadib et al., 2023; Hitosugi et al., 2004; Kornblau and El-Annan, 2019; Su et al., 2012). Furthermore, traditional FA has a limited field of view, typically capturing a limited area of the posterior pole, thereby missing peripheral retinal pathology that is critical in many retinal diseases (Temkar et al., 2019; Manivannan et al., 2005). Recent technological advancements have led to the development of ultra-wide-field (UWF) imaging systems, which have revolutionized retinal assessment. These systems enable comprehensive visualization of up to 200^o^ field of the retina, including the far periphery, facilitating the detection of peripheral pathologies that were previously difficult to document (Lip et al., 2020; Tsui et al., 2013; Friberg et al., 2008). The integration of UWF imaging with FA has significantly enhanced our ability to evaluate a wide spectrum of retinal diseases.

Orally administered fluorescein angiography (oral FA) combined with ultra-wide-field imaging represents a significant innovation in retinal imaging. This technique involves oral administration of fluorescein dye followed by UWF imaging to provide detailed visualization of retinal vasculature and pathology (Marziali et al., 2022; Manoharan et al., 2017). Compared to conventional IVFA, oral FA offers several potential advantages: reduced risk of adverse reactions associated with intravenous injection, improved patient comfort and acceptance (particularly in pediatric populations and needle-phobic individuals), and the logistical simplicity of avoiding venipuncture. While the application of IVFA with UWF imaging for evaluating various fundus disorders has been well documented, studies focusing on oral FA remain limited. Previous studies have primarily explored this technique in pediatric populations (Elhusseiny et al., 2023; Marmoy et al., 2022), with few reports systematically evaluating its utility across a broad age spectrum or comparing its performance across different retinal disease categories. Furthermore, comprehensive assessments of image quality, circulation timing, and safety profiles of oral FA in diverse patient populations are lacking.

This study aims to systematically evaluate the clinical utility of oral FA by: 1) Assess whether oral FA provides diagnostic image quality comparable to that of IVFA across a broad spectrum of fundus diseases; 2) Evaluate its feasibility and performance across different disease categories and age groups; 3) Document the pharmacokinetic profile of oral FA, including circulation time and phase visualization; 4) Determine the safety of oral FA. By systematically analyzing these objectives, we seek to establish the clinical utility and limitations of oral FA and provide evidence for its potential role as a viable alternative to conventional IVFA in the management of ocular diseases.

## 2 Patients and methods

### 2.1 Patients ethics

The patients with various fundus diseases who received fluorescence vascular examination were enrolled consecutively in this study between March 2023 and June 2024 at Shenzhen Eye Hospital, China. This study follows the Helsinki Declaration and has been approved by the Medical Ethics Committee of the Shenzhen Ophthalmology Center (MR-44-23-002037). Oral FA involves the use of fluorescein sodium, and it is necessary to explain the potential risks and benefits to patients or their guardians to ensure informed decision-making. Therefore, we explained in detail the advantages and disadvantages of oral FA to the patients or their guardians and obtained informed consent (including parental/guardian consent for minors) from all participants.

### 2.2 Procedures

Using tropicamide (1%) to dilate the patient’s pupils before imaging, while loratadine and vitamin B6 were taken orally. Ultra-wide-field scanning laser ophthalmoscopy (Optos® California P200DTx, Optos plc, United Kingdom) was used for retinal angiograms of oral FA.

Previous studies have shown that the recommended dose of sodium fluorescein to generate an optimal oral angiogram was 20–30 mg/kg (Fung et al., 2014; Amram et al., 2018; Ali et al., 2018; Sugimoto et al., 2014). In this study, approximately 30 mg/kg of 20% fluorescein sodium (Xlateming®, Baiyunshan Mingxing, Guangzhou, China) was mixed with 10 mL of water and administered orally to each patient.

The imaging system timer was started once the patients had ingested the full dose of fluorescein sodium, as precise timing is critical to capture the optimal phases of fluorescence, ensuring high-quality imaging of the retinal vasculature. Images were taken every 1 s during the early arteriovenous phase and then every 5 min until the late phase was reached.

### 2.3 Definition of circulation times

**FIGURE 1:**
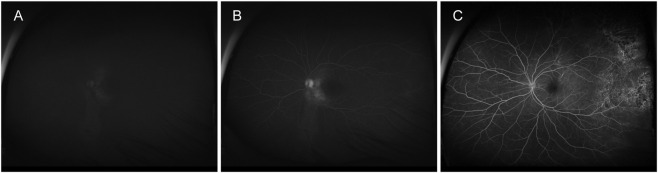
The representative images of different circulation time points. **(A)** The representative images of first appearance. **(B)** The representative images of detailed visualization. **(C)** The representative images of optimal visualization.

The following key circulation time points were systematically recorded after oral fluorescein ingestion. The definitions were established prior to data analysis to ensure objectivity and reproducibility: 1) First appearance time: Defined as the time interval between oral intake and the first appearance of sodium fluorescein image on the optic disc. 2) Detailed visualization time: Defined as the time interval between oral intake and the visualization of retinal capillaries, macular arch or tertiary branch vessels. 3) Optimal visualization time: Defined as the time interval between oral intake and the frame exhibiting the peak fluorescence intensity of the entire fundus, resulting in the maximum contrast between the retinal vasculature and the background choroidal flush. All time points were determined by consensus between experienced graders who reviewed the entire angiographic sequence for each eye. Representative images of these key time points are shown in Figure 1.

### 2.4 Image analysis

To quantitatively evaluate the utility of oral FA, the images were anonymized and randomized. Then, four experienced retinal specialists performed quality scoring, and all the scores were independently conducted. Only peak fluorescence filling phase images were provided for evaluation to reduce bias. Three parameters were used to score the images, modified from previously used methods (Amador-Patarroyo et al., 2020; Squirrell et al., 2005; Sawyer et al., 2024). The three parameters were: (Ⅰ) branch retinal vessel visualization, (Ⅱ) foveal avascular zone (FAZ) visualization, and (Ⅲ) clinically important findings, such as the presence of microaneurysms, neovascularization, leakage, visualization of nonperfusion areas, or macular edema. Three parameters score were as follows.

1. 0 points: first-order branches are seen; 1 point: second-order branches are seen; 2 points: third-order branches are seen.
2. 0 points: FAZ is impossible to judge; 1 point: FAZ is seen but not clearly; 2 points: FAZ is seen clearly.
3. 0 points: impossible to judge; 1 point: seen but not clearly; 2 points: seen clearly.

**FIGURE 2:**
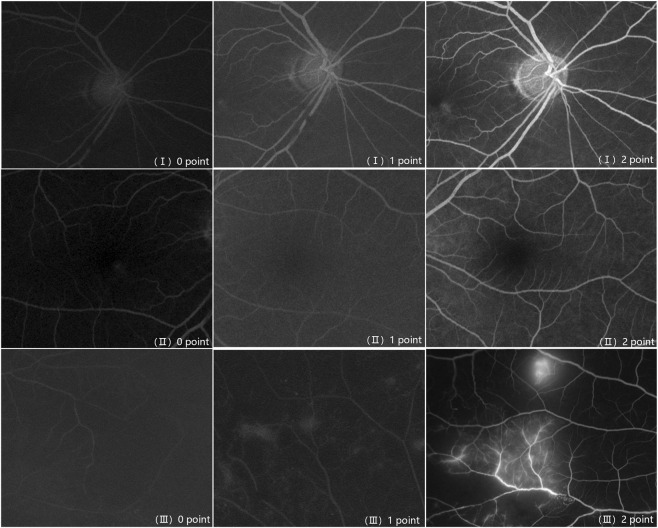
Representative images of parameters I, II and III.

Representative images with scores for each of these parameters are shown in Figure 2. Representative images of different phases of oral FA are shown in Supplementary Figure S1.

We used the sum of three parameter to evaluate image quality, which was modified from the methods previously used by Sayaka et al. (Yamao et al., 2021). The classification thresholds for image quality were established based on the consensus of all four retinal specialists, who independently determined the minimum scores required for clinical utility. Specifically, they agreed that parameter (I) must score 2 points (i.e., third-order branches visible), and parameters (II) and (III) must each score at least 1 point (i.e., FAZ and clinically important findings are discernible). These criteria correspond to a minimum total score of 4 points, which was defined as the threshold for clinically useful images. A total score of 5 or more points was considered high quality, as it indicates that at least one additional parameter achieved a higher score, further enhancing diagnostic confidence. Therefore, we classified the images into the following three grades to assess image quality: images with 5 points or more were defined as high quality. Clinically useful quality was required for a total score of 4 or more. Total scores between 4 and 5 points were considered moderate quality, and less than 4 points were considered poor quality.

### 2.5 Statistical analysis

Statistical analysis was performed using SPSS (version 27.0) and Prism 10 (GraphPad). The normality of data distribution was assessed using the Shapiro-Wilk test, and the homogeneity of variances was evaluated using Levene’s test. For comparisons among three or more groups, if the data met both assumptions of normality and homogeneity of variance, one-way analysis of variance (ANOVA) followed by Tukey’s post-hoc test was applied. If the assumption of homogeneity of variance was violated, Welch’s ANOVA with Games-Howell post-hoc test was used. For comparisons between two independent groups, unpaired Student’s t-test or the Mann-Whitney U test was used based on the normality of the data. The Kruskal–Wallis test with Dunn’s post-hoc test was employed for multi-group comparisons when data were not normally distributed. Statistical significance was set at *P* < 0.05. Inter-observer reliability for the image quality scores was assessed using a two-way random-effects, absolute agreement, intraclass correlation coefficient (ICC).

## 3 Results

### 3.1 Patients characteristics

A total of 382 patients (676 eyes) aged between 4 and 83 years were recruited for this study, consisting of 164 women and 218 men. According to age, patients are divided into three age groups: 80 patients (20.9%) with 153 (22.6%) eyes were under 18 years old; 245 (64.1%) patients with 431 (63.8%) eyes were between 18 and 60 years old; and 57 (15.0%) patients with 92 (13.6%) eyes were over 60 years old. According to the pathology and location of fundus diseases, clinical diagnoses were divided into five categories: retinal vascular diseases (including central retinal vein occlusion, branch retinal vein occlusion, diabetic retinopathy, familial exudative vitreoretinopathy, Coats’ disease, retinopathy of prematurity, and retinal vasculitis), chorioretinitis (including uveitis, choroiditis, and Vogt-Koyanagi-Harada Syndrome), retinal degenerative diseases (including retinitis pigmentosa, retinal detachment, and peripheral retinal degeneration), macular diseases (including central serous chorioretinopathy, age-related macular degeneration, central exudative chorioretinitis, epiretinal membrane, macular edema or hole, and retinoschisis), and optic nerve diseases (including anterior ischemic optic neuropathy, optic neuritis, and glaucoma). 131 (34.3%) patients with 220 (32.5%) eyes for retinal vascular diseases; 91 (23.8%) patients with 168 (24.9%) eyes for chorioretinitis; 75 (19.6%) patients with 146 (21.6%) eyes for retinal degenerative diseases; 59 (15.4%) patients with 93 (13.8%) eyes for macular diseases; and 26 (6.8%) patients with 49 (7.2%) eyes for optic nerve diseases. The characteristics of the patients in different groups are shown in Table 1.

**TABLE 1:** Patients characters in different groups.

Group	Patients (%)	Eyes (%)	Years (mean ± SD)	Male	Female
<18	80 (20.9%)	153 (22.6%)	10.650 ± 3.562	40	40
18–60	245 (64.1%)	431 (63.8%)	38.996 ± 11.247	151	94
>60	57 (15.0%)	92 (13.6%)	67.175 ± 6.024	27	30
Total	382	676	​	218	164
Retinal vascular diseases	131 (34.3%)	220 (32.5%)	41.450 ± 21.389	80	51
Chorioretinitis	91 (23.8%)	168 (24.9%)	32.473 ± 18.439	44	47
Retinal degenerative diseases	75 (19.6%)	146 (21.6%)	28.627 ± 16.978	37	38
Macular disease	59 (15.4%)	93 (13.8%)	43.712 ± 13.340	43	16
Optic nerve diseases	26 (6.8%)	49 (7.2%)	43.231 ± 16.985	14	12
Total	382	676	​	218	164

### 3.2 Image quality analysis

**FIGURE 3:**
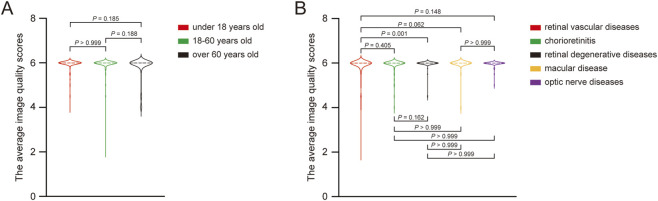
The average image quality scores pairwise comparison of different groups. **(A)** The average image quality scores pairwise comparison of different age groups. **(B)** The average image quality scores pairwise comparison of different fundus diseases.

Image quality was assessed using three parameters. The final score for each image was calculated as the mean of the scores provided by the four independent retina specialists. The inter-observer reliability for the total image quality score was good. The ICC for single measures was 0.863 (95% CI: 0.847 - 0.889) for right eyes and 0.869 (95% CI: 0.841 - 0.884) for left eyes. Among the 382 cases (676 eyes) that underwent oral FA, 374 cases (662 eyes) were classified as high quality, 7 cases (12 eyes) as moderate quality, and 1 case (2 eyes) as low quality. Clinically useful images were obtained in 381 out of 382 cases (99.7%). One case of central retinal vein occlusion was classified as low quality due to vitreous hemorrhage obscuring the fundus. As shown in Table 2, the average image quality scores (AIQS) were 5.904 ± 0.326 in the under 18 years age group, 5.893 ± 0.415 in the 18–60 years group, and 5.796 ± 0.531 in the over 60 years group. When grouped by disease, the AIQS was 5.807 ± 0.565 for retinal vascular diseases, 5.881 ± 0.374 for chorioretinitis, 5.950 ± 0.243 for retinal degenerative diseases, 5.917 ± 0.348 for macular diseases, and 5.959 ± 0.164 for optic nerve diseases. No statistically significant differences were found among the different age groups (all *P* > 0.05). Among the disease categories, only retinal vascular diseases and retinal degenerative diseases show a statistically significant difference (*P* = 0.001, Figure 3).

**TABLE 2:** AIQS of different groups.

Group	AIQS (mean ± SD)
<18	5.904 ± 0.326
18–60	5.893 ± 0.415
>60	5.796 ± 0.531
Retinal vascular diseases	5.807 ± 0.565
Chorioretinitis	5.881 ± 0.374
Retinal degenerative diseases	5.950 ± 0.243
Macular disease	5.917 ± 0.348
Optic nerve diseases	5.959 ± 0.164

AIQS, average image quality scores.

**FIGURE 4:**
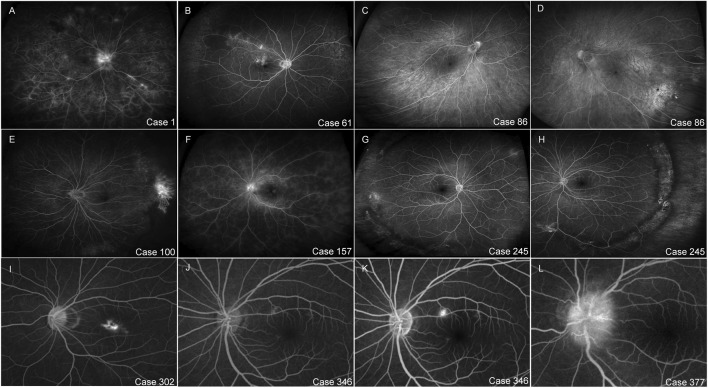
The images of representative cases. **(A)** Case 1: Diabetic retinopathy, neovascularization at the optic disc, non-perfusion areas. **(B)** Case 61: BRVO, peripheral non-perfusion area of superior temporal retina. **(C,D)** Case86: ROP, brush-like changes in peripheral retinal vessels in both eyes. **(E)** Case 100: FEVR, the peripheral retinal vessels on the nasal side of the right eye exhibited brush-like alterations and neovascularization observed clearly anterior to the crests. **(F)** Case 157: Uveitis, reflected fluorescence leakage from the retinal capillaries. **(G,H)** Case 245: Peripheral retinal degeneration, peripheral retinal degeneration areas and holes in both eyes. **(I)** Case 302: Central exudative chorioretinitis, macular choroidal neovascularization. **(J,K)** Case 346: Central serous chorioretinopathy, macular fluorescence leakage points expanded gradually. **(L)** Case 377: Optic neuropathy, fluorescence leakage of optic disc.

For images with clinically important findings, such as microaneurysms, neovascularization, leakage, or significant nonperfusion areas visible in the temporal peripheral retina, all observers gave the maximum score. These findings were clearly visualized, except in the low-quality image caused by retinal obstruction. The characteristics and images of representative cases are displayed in Table 3 and Figure 4, respectively.

**TABLE 3:** The characteristics of representative cases.

Case no.	Sex	Year	Diagnosis	AIQS OD/OS
1	Female	34	Diabetic retinopathy	6/6
61	Female	79	BRVO	6/Normal
86	Female	6	ROP	6/6
100	Male	22	FEVR	6/6
157	Female	23	Uveitis	6/6
245	Female	18	Peripheral retinal degeneration	6/6
302	Male	19	Central exudative chorioretinitis	Normal/6
346	Male	33	Central serous chorioretinopathy	Normal/6
377	Female	27	Optic neuropathy	6/6

BRVO, branch retinal vein occlusion; ROP, retinopathy of prematurity; FEVR, familial exudative vitreoretinopathy; AIQS, average image quality scores.

### 3.3 Phase wise vascular visualization following oral intake

**FIGURE 5:**
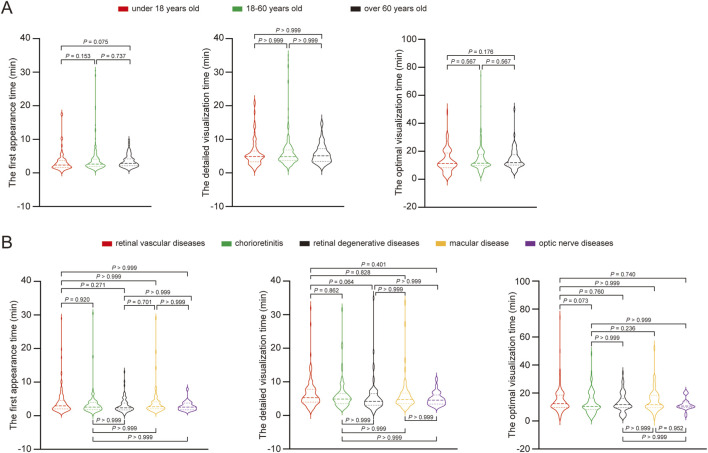
The circulation times pairwise comparison of different groups. **(A)** The circulation times pairwise comparison of different age groups. **(B)** The circulation times pairwise comparison of different fundus diseases.

As shown in Table 4, in the under 18 years group, the first appearance time was 3.213 ± 2.958 min, detailed visualization time was 5.804 ± 3.990 min, and optimal visualization time was 12.996 ± 8.109 min. In the 18–60 years group, these times were 3.702 ± 3.846 min, 6.100 ± 4.831 min, and 13.802 ± 7.889 min, respectively. In the over 60 years group, the times were 3.436 ± 1.861 min, 5.722 ± 2.886 min, and 14.524 ± 7.471 min, respectively. When categorized by disease, the first appearance time was 3.920 ± 3.679 min for retinal vascular diseases, 3.362 ± 3.566 min for chorioretinitis, 3.061 ± 2.162 min for retinal degenerative diseases, 4.008 ± 4.452 min for macular diseases, and 2.859 ± 1.374 min for optic nerve diseases. The detailed visualization time were 6.408 ± 4.246 min, 5.981 ± 4.337 min, 5.576 ± 4.618 min, 6.204 ± 5.327 min, and 4.956 ± 1.964 min, respectively. The optimal visualization time were 14.781 ± 8.888 min, 12.761 ± 7.738 min, 13.003 ± 6.762 min, 14.677 ± 7.997 min, and 11.973 ± 4.248 min, respectively. Whether grouped by age or disease category, there were no statistically significant differences in the recorded times among the different subgroups within each grouping category (Figure 5).

**TABLE 4:** The first appearance time, detailed visualization time and optimal visualization time of different groups.

Group	First appearance time (mean ± SD)	Detailed visualization time (mean ± SD)	Optimal visualization time (mean ± SD)
<18	3.213 ± 2.958	5.804 ± 3.990	12.996 ± 8.109
18–60	3.702 ± 3.846	6.100 ± 4.831	13.802 ± 7.889
>60	3.436 ± 1.861	5.722 ± 2.886	14.524 ± 7.471
Retinal vascular diseases	3.920 ± 3.679	6.408 ± 4.246	14.781 ± 8.888
Chorioretinitis	3.362 ± 3.566	5.981 ± 4.337	12.761 ± 7.738
Retinal degenerative diseases	3.061 ± 2.162	5.576 ± 4.618	13.003 ± 6.762
Macular disease	4.008 ± 4.452	6.204 ± 5.327	14.677 ± 7.997
Optic nerve diseases	2.859 ± 1.374	4.956 ± 1.964	11.973 ± 4.248

### 3.4 Adverse events

Six patients experienced nausea, and two patients had mild skin rash, which resolved spontaneously after about an hour. The characteristics of adverse event cases are displayed in Table 5. In other cases, including 6 patients with a history of fluorescein allergy during IVFA, there were no adverse events after oral FA. The incidence of adverse drug reactions was 2.1%.

**TABLE 5:** The characteristics of adverse event cases.

Case no.	Sex	Year	Adverse event	Occurrence time after oral fluorescent sodium	Treatment method and outcome
14	Male	56	Mild skin rash	55 min	Symptoms relieved after taking oral loratadine
63	Male	49	Mild nausea and vomiting reaction	1 min	Deep breath, symptoms relieved after vomiting
81	Female	46	Mild nausea and vomiting reaction	1 min	Deep breath, symptoms relieved after vomiting
96	Female	32	Mild nausea and vomiting reaction	3 min	Deep breath, symptoms relieved after vomiting
126	Male	9	Mild nausea and vomiting reaction	2 min	Deep breath, symptoms relieved after vomiting
178	Male	27	Mild nausea and vomiting reaction	1 min	Deep breath, symptoms relieved after vomiting
223	Female	15	Mild nausea and vomiting reaction	2 min	Deep breath, symptoms relieved after vomiting
256	Male	21	Mild skin rash	5 min	Symptoms relieved after taking oral loratadine

## 4 Discussion

Fluorescein angiography (FA) is a key technique for diagnosing retinal diseases (Lv et al., 2023). However, the risk of severe allergic reactions associated with IVFA remains a significant concern in clinical practice (Occhiutto et al., 2012; Ghaleb et al., 2025). Oral FA substantially reduces this risk, yet its widespread application has long been limited by perceived poor image quality (Jiang et al., 2022; Nayak and Ghose, 1987). This study, utilizing ultra-wide-field scanning laser ophthalmoscopy and an appropriate dose of sodium fluorescein, systematically evaluated the image quality, circulation time, and safety of oral FA across different age groups and various retinal diseases. The results demonstrate that oral FA is suitable not only for children but also generates high quality, diagnostically valuable images in adult and elderly patients, serving as a safe, effective, and convenient alternative to IVFA.

Previous research on oral FA has primarily focused on pediatric populations, especially preschoolers and school-aged children (Yamao et al., 2021; Conner et al., 2023; Ling et al., 2022). This is mainly due to lower acceptance and poorer cooperation with venipuncture in children, whereas the oral route is more readily accepted by both young patients and their parents. For instance, Yamao et al. showed that oral FA combined with ultra-wide-field imaging effectively evaluated key findings such as the foveal avascular zone (FAZ) and neovascularization in children (Yamao et al., 2021). Although Hara et al. demonstrated the feasibility of oral FA in 1787 patients, systematic subgroup comparisons were not performed (Hara et al., 1998). However, these studies lacked systematic evaluation in adult and elderly populations. Our study enrolled 382 patients (676 eyes), covering a wide age range from 4 to 83 years, and performed stratified analysis based on age and disease type, addressing the shortcomings of previous studies with a single population and inadequate stratification. It not only confirmed the advantages of oral FA in children but also, for the first time, systematically compared image quality and circulation characteristics in adult and elderly patients, expanding the applicable scope of oral FA.

This study shows that high quality angiograms can be obtained with oral FA in the vast majority of cases. Overall, 374 out of 382 cases (662 out of 676 eyes) were classified as high quality. Clinically useful images were obtained in 381 cases (99.7%), with only one case yielding low-quality images due to vitreous hemorrhage. When stratified by age, no statistically significant differences were found in the average image quality scores among the different age groups (*P* > 0.05), suggesting that oral FA maintains stable image quality across all age ranges. This finding is important considering that earlier studies focused predominantly on pediatric populations and rarely included adults or the elderly. When grouped by disease type, all groups maintained high image quality scores, yet a statistically significant difference was observed between retinal vascular diseases and retinal degenerative diseases. This discrepancy may be related to different pathological and physiological changes in various diseases. Retinal degenerative diseases, such as retinitis pigmentosa, are primarily characterized by a progressive loss of photoreceptors and retinal pigment epithelium (RPE) (Viktoria et al., 2023; Xiaoqian et al., 2022). This often leads to attenuation of retinal vessels and widespread RPE atrophy. The subsequent reduction in both vascular density and the masking effect of the RPE may result in a clearer, higher-contrast angiographic image, as the fluorescent dye circulates through a less crowded and less obscured vascular bed. Conversely, retinal vascular diseases, including diabetic retinopathy and retinal vein occlusions, are frequently associated with pathological changes that directly impair image clarity. Media opacities such as vitreous hemorrhage, intraretinal edema, and hard exudates can scatter and attenuate the fluorescent signal. Furthermore, the presence of extensive capillary dropout or non-perfusion can reduce the overall fluorescence intensity in large areas of the retina. These factors collectively contribute to the relatively lower, though still diagnostically adequate, image quality scores in this category. Previous oral FA studies (Conner et al., 2023; Ling et al., 2022), limited primarily to children, could not perform such cross-disease comparisons. Therefore, our data provide new insights into the performance of oral FA across different patient populations.

This study also systematically recorded three key time points after oral fluorescein administration: first appearance time, detailed visualization time, and optimal visualization time. The results showed no statistically significant differences in these time points among the different age groups (*P* > 0.05). This indicates that even in elderly patients, the absorption and circulation of fluorescein have not shown significant delay, further supporting the feasibility of oral FA across the entire age spectrum. Among disease groups, the optic nerve diseases group had the shortest circulation times at all phases, while the retinal vascular diseases group was slightly longer, possibly related to the fact that the latter often accompanies blood circulation disorders. However, no statistical differences were reached between groups, indicating stable pharmacokinetic characteristics of oral FA across different retinal diseases.

Compared to IVFA, oral FA has several advantages. Firstly, the incidence of adverse reactions to oral FA is usually lower than that of IVFA (Garcia et al., 1999; Kelley and Kincaid, 1979; Watson and Rosen, 1990). No serious adverse events have been reported in previous oral FA studies (Barteselli et al., 2013; Kwan et al., 2006). For example, Hara et al. assessed the safety of oral FA in 1787 patients and found only 31 (1.7%) experienced minor symptoms like itching or nausea, with no severe adverse reactions observed (Kelley and Kincaid, 1979). In our study, mild adverse reactions occurred in 8 patients (2.1%), including 2 cases of mild rash and 6 cases of nausea, all of which resolved spontaneously within 1 hour. No severe allergic reactions were observed. Notably, 6 patients in this study had a history of fluorescein allergy, but no adverse reactions were observed after taking oral FA, further demonstrating the safety of oral FA. Compared to IVFA, oral FA significantly reduces the risk of allergies, especially suitable for patients with a history of allergies. Secondly, oral FA does not require needle venipuncture, making it more acceptable to patients, especially minors. In this study, all minor patients completed the oral FA and obtained high quality contrast images. Therefore, oral FA is suitable for patients who cannot tolerate venipuncture or cannot cooperate, especially pre-school and school-age children.

Despite its advantages, oral FA has certain limitations. First, it struggles to capture images of the early ocular circulation phases, such as the arterial phase. In this study, the arterial phase was absent in almost all cases. The slower rise in plasma sodium fluorescein concentration after oral administration compared to intravenous injection nearly prevents the capture of early retinal arterial perfusion. This makes oral FA less suitable for conditions requiring observation of early retinal vascular filling, such as central retinal vein occlusion. However, for most retinal diseases, including diabetic retinopathy, uveitis, and macular diseases, it provides sufficient diagnostic information. Second, the bitter taste of the sodium fluorescein solution may be difficult for some patients to tolerate, potentially preventing successful ingestion. Mixing the dye with sugar or fruit juice can help improve its taste.

This study also has several limitations. First, although the sample size is larger than in previous studies, the number of cases in certain subgroups (such as the optic nerve diseases) remains relatively small. This may limit the statistical power to detect subtle differences in image quality or circulation timing within these specific disease categories. Future studies with larger, more balanced cohorts are needed to confirm these subgroup findings. Second, while we included a broad spectrum of fundus diseases, certain rare or complex conditions may not have been sufficiently represented. Expanding the range of diseases in future investigations would help to further validate the generalizability of oral FA in diverse clinical scenarios. Third, this was a single-center study and the findings may be influenced by local patient demographics, equipment-specific factors, or institutional practices. Further multi-center studies involving diverse geographic and clinical settings would help to corroborate our results and strengthen the external validity of oral FA as a widely applicable diagnostic tool.

## 5 Conclusion

Oral FA is a safe, well-tolerated, and clinically effective imaging modality for evaluating retinal diseases across different age groups. Our results confirm its utility not only in children but also in adults and the elderly. The ability to obtain high quality, diagnostically adequate images without the need for intravenous access makes oral FA a promising alternative to conventional IVFA, particularly in settings where patient comfort, safety, and resource efficiency are prioritized. In summary, our study systematically validates the safety and applicability of oral FA across different ages and diseases in a large cohort, demonstrating its promise as a viable alternative to IVFA for specific populations such as pediatric, needle-phobic, or allergy-prone patients.

## Data Availability

The original contributions presented in the study are included in the article/Supplementary Material, further inquiries can be directed to the corresponding authors.

## References

[B1] Ahmed M. Syrine B. M. Nadia B. A. Anis M. Karim Z. Mohamed G. (2021). Optical coherence tomography angiography features of macular neovascularization in wet age-related macular degeneration: a cross-sectional study. Ann. Med. Surg. (Lond). 70 (0), 102826. 10.1016/j.amsu.2021.102826 34540215 PMC8435926

[B2] Ali S. M. A. Khan I. Khurram D. Kozak I. (2018). Ultra-widefield angiography with oral fluorescein in pediatric patients with retinal disease. JAMA Ophthalmol. 136 (5), 593–594. 10.1001/jamaophthalmol.2018.0462 29621363 PMC6583855

[B3] Amador-Patarroyo M. J. Lin T. Meshi A. Dans K. C. Chen K. Borooah S. (2020). Identifying the factors for improving quality of oral fluorescein angiography. Br. J. Ophthalmol. 104 (4), 504–508. 10.1136/bjophthalmol-2019-314187 31272951

[B4] Amram A. L. Makkouk F. Pace S. T. Kellogg C. Elkeeb A. (2018). Sublingual/transmucosal fluorescein angiography. Ophthalmol. Retina 2 (9), 980–982. 10.1016/j.oret.2018.04.001 31047232

[B5] Barteselli G. Chhablani J. Lee S. N. Wang H. El Emam S. Kozak I. (2013). Safety and efficacy of oral fluorescein angiography in detecting macular edema in comparison with spectral-domain optical coherence tomography. Retina 33 (8), 1574–1583. 10.1097/IAE.0b013e318285cd84 23584697 PMC3804067

[B6] Bulson R. Faridi A. (2017). Normotensive glaucoma Follow-Up with incidental finding of choroidal neovascular membrane: a teaching case report. Optom. Educ. 42 (17), 16–22. 37287474 PMC10246759

[B7] Chen X. Zhu W. Li X. (2022). OCT macular volume as a predictor of vascular leakage in uveitis. Ophthalmol. Ther. 11 (5), 1913–1924. 10.1007/s40123-022-00558-z 35978263 PMC9437176

[B8] Conner E. A. Eldib A. Hiasat J. G. Pihlblad M. S. Errera M. H. Chhablani P. P. (2023). Pediatric oral fluorescein angiography: a retrospective review from a single institution. J. AAPOS 27 (4), 191 e1–e6. 10.1016/j.jaapos.2023.06.004 37507064

[B9] Ebrahimiadib N. Hadavand Mirzaei S. Riazi-Esfahani H. Amini M. (2023). Renal function following fluorescein angiography in diabetic patients with chronic kidney disease. J. Ophthalmic Vis. Res. 18 (2), 170–174. 10.18502/jovr.v18i2.13183 37181615 PMC10172804

[B10] Elhusseiny A. M. Fong J. W. Hsu C. Grigorian F. Grigorian A. P. Soliman M. K. (2023). Oral fluorescein angiography for the diagnosis of papilledema *versus* pseudopapilledema in children. Am. J. Ophthalmol. 245 (0), 8–13. 10.1016/j.ajo.2022.08.020 36084685

[B11] Friberg T. R. Gupta A. Yu J. Huang L. Suner I. Puliafito C. A. (2008). Ultrawide angle fluorescein angiographic imaging: a comparison to conventional digital acquisition systems. Ophthalmic Surg. Lasers Imaging 39 (4), 304–311. 10.3928/15428877-20080701-06 18717436

[B12] Fung T. H. Muqit M. M. Mordant D. J. Smith L. M. Patel C. K. (2014). Noncontact high-resolution ultra-wide-field oral fluorescein angiography in premature infants with retinopathy of prematurity. JAMA Ophthalmol. 132 (1), 108–110. 10.1001/jamaophthalmol.2013.6102 24201455

[B13] Garcia C. R. Rivero M. E. Bartsch D. U. Ishiko S. Takamiya A. Fukui K. (1999). Oral fluorescein angiography with the confocal scanning laser ophthalmoscope. Ophthalmology 106 (6), 1114–1118. 10.1016/S0161-6420(99)90264-6 10366079

[B14] Ghaleb R. Sallam A. B. Grigorian F. Phillips P. H. Elhusseiny A. M. (2025). Oral fluorescein angiography in pediatric ophthalmology. Surv. Ophthalmol. 70 (5), 849–858. 10.1016/j.survophthal.2025.03.005 40089030

[B15] Hans A. Narang S. Sindhu M. Jain S. Chawla D. (2022). Fundus fluorescein angiography in retinopathy of prematurity. Eye (Lond). 36 (8), 1604–1609. 10.1038/s41433-021-01694-9 34290444 PMC9307517

[B16] Hara T. Inami M. Hara T. (1998). Efficacy and safety of fluorescein angiography with orally administered sodium fluorescein. Am. J. Ophthalmol. 126 (4), 560–564. 10.1016/s0002-9394(98)00112-3 9780101

[B17] He J. Wang J. Han Z. Ma J. Wang C. Qi M. (2023). An interpretable transformer network for the retinal disease classification using optical coherence tomography. Sci. Rep. 13 (1), 3637. 10.1038/s41598-023-30853-z 36869160 PMC9984386

[B18] Hitosugi M. Omura K. Yokoyama T. Kawato H. Motozawa Y. Nagai T. (2004). An autopsy case of fatal anaphylactic shock following fluorescein angiography: a case report. Med. Sci. Law 44 (3), 264–265. 10.1258/rsmmsl.44.3.264 15296251

[B19] Ji Y. Ji Y. Liu Y. Zhao Y. Zhang L. (2023). Research progress on diagnosing retinal vascular diseases based on artificial intelligence and fundus images. Front. Cell Dev. Biol. 11 (0), 1168327. 10.3389/fcell.2023.1168327 37056999 PMC10086262

[B20] Jiang Z. Sun L. Hou A. Zhang T. Lai Y. Huang L. (2022). Oral fluorescein angiography with ultra-wide-field scanning laser ophthalmoscopy in pediatric patients precis: oral fluorescein angiography in children. J. Clin. Med. 11 (18), 5421. 10.3390/jcm11185421 36143067 PMC9500735

[B21] Kelley J. S. Kincaid M. (1979). Retinal fluorography using oral fluorescein. Arch. Ophthalmol. 97 (12), 2331–2332. 10.1001/archopht.1979.01020020547007 518385

[B22] Kornblau I. S. El-Annan J. F. (2019). Adverse reactions to fluorescein angiography: a comprehensive review of the literature. Surv. Ophthalmol. 64 (5), 679–693. 10.1016/j.survophthal.2019.02.004 30772364

[B23] Kwan A. S. Barry C. McAllister I. L. Constable I. (2006). Fluorescein angiography and adverse drug reactions revisited: the lions eye experience. Clin. Exp. Ophthalmol. 34 (1), 33–38. 10.1111/j.1442-9071.2006.01136.x 16451256

[B24] Li Z. Han Y. Yang X. (2023). Multi-fundus diseases classification using retinal optical coherence tomography images with swin transformer V2. J. Imaging 9 (10), 203. 10.3390/jimaging9100203 37888310 PMC10607340

[B25] Ling X. C. Chou H. D. Liu L. Wang N. K. Lai C. C. Chen K. J. (2022). Comparison between oral and intravenous ultrawide-field fluorescein angiography in the clinical Follow-up of children with a history of retinopathy of prematurity or prematurity. Retina 42 (7), 1330–1337. 10.1097/IAE.0000000000003451 35723921

[B26] Lip P. L. Kolli H. Trivedi D. (2020). Ultra-widefield fluorescein angiographic patterns, retinal microvascular anomalies and retinal ischemic index in branch retinal vein occlusions with established retinal neovascularization. Clin. Ophthalmol. 14 (0), 2965–2974. 10.2147/OPTH.S272064 33061282 PMC7534860

[B27] Lv M. Li T. Li Y. (2023). Clinical application of optical coherence tomography angiography in diabetic macular edema. Afr. Health Sci. 23 (2), 484–489. 10.4314/ahs.v23i2.56 38223604 PMC10782371

[B28] Manivannan A. Plskova J. Farrow A. McKay S. Sharp P. F. Forrester J. V. (2005). Ultra-wide-field fluorescein angiography of the ocular fundus. Am. J. Ophthalmol. 140 (3), 525–527. 10.1016/j.ajo.2005.02.055 16139004

[B29] Manoharan N. Pecen P. E. Cherof A. M. Oliver S. C. N. Palestine A. G. (2017). Comparison of oral Versus intravenous fluorescein widefield angiography in ambulatory pediatric patients. J. Vitreoretin. Dis. 1 (3), 191–196. 10.1177/2474126417705256

[B30] Marmoy O. R. Henderson R. H. Ooi K. (2022). Recommended protocol for performing oral fundus fluorescein angiography (FFA) in children. Eye (Lond). 36 (1), 234–236. 10.1038/s41433-020-01328-6 33323986 PMC8727556

[B31] Marziali E. Testi I. MacPhee B. Ibanez P. Allen M. Dahlmann-Noor A. (2022). A prospective evaluation of adverse events occurring in children undergoing fundus fluorescein and indocyanine green angiography. Eye (Lond). 36 (9), 1837–1839. 10.1038/s41433-022-01951-5 35094031 PMC9391385

[B32] Nayak B. K. Ghose S. (1987). A method for fundus evaluation in children with oral fluorescein. Br. J. Ophthalmol. 71 (12), 907–909. 10.1136/bjo.71.12.907 3426997 PMC1041342

[B33] Noh M. Kim Y. Zhang H. Kim H. Bae C. R. Lee S. (2023). Oral administration of CU06-1004 attenuates vascular permeability and stabilizes neovascularization in retinal vascular diseases. Eur. J. Pharmacol. 939 (0), 175427. 10.1016/j.ejphar.2022.175427 36509133

[B34] Occhiutto M. L. Freitas F. R. Maranhao R. C. Costa V. P. (2012). Breakdown of the blood-ocular barrier as a strategy for the systemic use of nanosystems. Pharmaceutics 4 (2), 252–275. 10.3390/pharmaceutics4020252 24300231 PMC3834913

[B35] Sawyer C. Huang L. C. Vincent J. Cabrera M. T. Herlihy E. Mustafi D. (2024). Diagnostic role of oral fluorescein angiography in pediatric ambulatory clinics. Ophthalmol. Retina 8 (2), 204–206. 10.1016/j.oret.2023.10.014 38707762 PMC11068305

[B36] Squirrell D. Dinakaran S. Dhingra S. Mody C. Brand C. Talbot J. (2005). Oral fluorescein angiography with the scanning laser ophthalmoscope in diabetic retinopathy: a case controlled comparison with intravenous fluorescein angiography. Eye (Lond). 19 (4), 411–417. 10.1038/sj.eye.6701513 15184968

[B37] Su Z. Ye P. Teng Y. Zhang L. Shu X. (2012). Adverse reaction in patients with drug allergy history after simultaneous intravenous fundus fluorescein angiography and indocyanine green angiography. J. Ocul. Pharmacol. Ther. 28 (4), 410–413. 10.1089/jop.2011.0221 22372690

[B38] Sugimoto M. Matsubara H. Miyata R. Matsui Y. Ichio A. Kondo M. (2014). Ultra-WideField fluorescein angiography by oral administration of fluorescein. Acta Ophthalmol. 92 (5), e417–e418. 10.1111/aos.12323 24529198

[B39] Temkar S. Azad S. V. Chawla R. Damodaran S. Garg G. Regani H. (2019). Ultra-widefield fundus fluorescein angiography in pediatric retinal vascular diseases. Indian J. Ophthalmol. 67 (6), 788–794. 10.4103/ijo.IJO_1688_18 31124488 PMC6552605

[B40] Tsui I. Bajwa A. Franco-Cardenas V. Pan C. K. Kim H. Y. Schwartz S. D. (2013). Peripheral fluorescein angiographic findings in fellow eyes of patients with branch retinal vein occlusion. Int. J. Inflam. 2013 (0), 464127. 10.1155/2013/464127 23607044 PMC3626174

[B41] Viktoria D. Akos L. Gergely S. Rovena S. Adrienn M. Andrea K.-V. (2023). Long-term effects of the pituitary-adenylate cyclase-activating polypeptide (PACAP38) in the adult mouse retina: microglial activation and induction of neural proliferation. Neurochem. Res. 48 (11), 3430–3446. 10.1007/s11064-023-03989-7 37466802 PMC10514177

[B42] Watson A. P. Rosen E. S. (1990). Oral fluorescein angiography: reassessment of its relative safety and evaluation of optimum conditions with use of capsules. Br. J. Ophthalmol. 74 (8), 458–461. 10.1136/bjo.74.8.458 2390518 PMC1042172

[B43] Xiaoqian D. Ryan L. Sin Yee L. Zheng Z. Jing W. Yizhi L. (2022). Global transcriptional and epigenetic reconfiguration during chemical reprogramming of human retinal pigment epithelial cells into photoreceptor-like cells. Cells 11 (19), 3146. 10.3390/cells11193146 36231108 PMC9564162

[B44] Yamao S. Tsujioka T. Takada R. Matsumoto F. Kusaka S. (2021). Utility of oral fluorescein angiography with ultra-widefield imaging system for evaluation of various retinal disorders. Retina 41 (6), 1338–1345. 10.1097/IAE.0000000000003011 33165297 PMC8140665

[B45] Yu T. Shao A. Wu H. Su Z. Shen W. Zhou J. (2025). A systematic review of advances in AI-Assisted analysis of fundus fluorescein Angiography (FFA) images: from detection to report generation. Ophthalmol. Ther. 14 (4), 599–619. 10.1007/s40123-025-01109-y 39982648 PMC11920566

