# Supplementary material for: Orally administered fluorescein angiography for ultra-wide-field imaging: is a safe and effective modality across different age groups and fundus diseases?
Source: Front Cell Dev Biol. 2026 Jan 8;13:1733128. doi: 10.3389/fcell.2025.1733128 (PMC12823843; doi:10.3389/fcell.2025.1733128)
Supplement: Supplementary file 1 [file DataSheet1.docx]

Supplementary materials


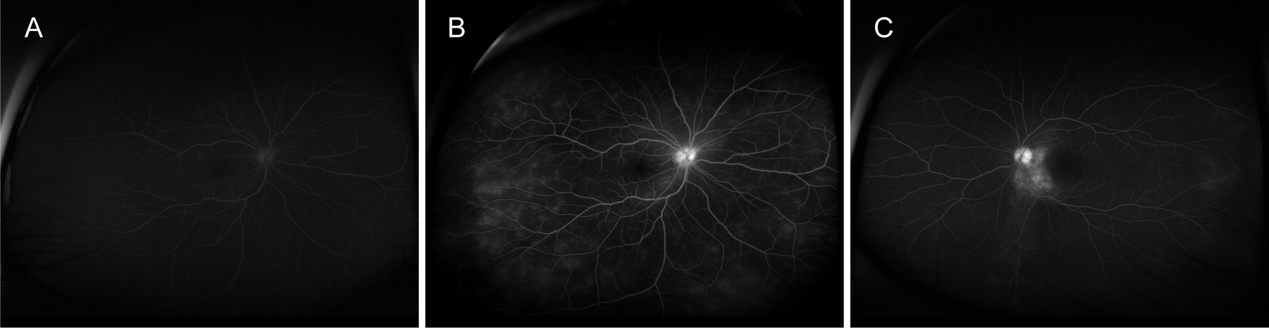


Figure S1: The representative images of different phases of oral FA. (A): The representative images of early phase. (B): The representative images of middle phase. (C): The representative images of late phase.
